# Experimental Biology

**Published:** 1911-03-11

**Authors:** 


### Experimental Biology.

It is perhaps possible, now that some time has elapsed since the republication of Mendel's paper and the fervour of enthusiasm has given way to cooler research, to examine the value of biological experiment, either in the narrow field of zoological study or in its wider application to sociological phenomena. There seems to be an unexpressed but nevertheless confident hope that the phenomena which are grouped together under the title "Heredity" will be found to be amenable to some universal and simple law. It is argued, and rightly, that the volume of Newton's "Principia" is dwarfed in comparison with the learned treatises of modern times, but the value of Newton's work is scarcely to be measured by the number of leaves. So therefore must it be with heredity. Since the pretensions of science *per se* have become so vast that she claims to direct the ancient department ruled over by philosophy, it has become the fashion to take every new discovery, particularly in the realm of biology, and endeavour to apply it as a fundamental principle to the problems of human life. Excellent as this utilitarian aspect of science is in itself, this method of hasty conclusion is to be deprecated. The little triumph won in the laboratory under perhaps artificially induced conditions is spread abroad by a Press eager for some sensational declaration, and the half-pondered theories of inexperienced seekers are passed from mouth to mouth as the last word of wisdom on that subject. Opposition is, we know, the breath of life to a theory which may indeed contain what we regard as truth, but such opposition is often based upon incorrect information supplied to the quasi-scientific journalist. Over-publication in scientific research is the curse of our present age. The reticence of Darwin, together with his spirit as a scientist, does not seem to have been handed down to the present generation, and, owing possibly to financial deficiences, a man is only too glad to publish rapidly and perpetually in order to keep his name before an unintelligent public. What value have experiments which involve the crossing of mice, of peas, of maize, of rabbits, and all the other selected examples of Mendelian heredity? They are certainly not valueless, but it is far too early at present to build any sociological structure upon them. To be told seriously by a writer that "broodiness" in hens is a heritable quality does not advance us much further unless at the same time we are told how this quality can be produced or dismissed at the will of the breeder, without the sudden appearance of some compensating but hitherto latent disqualification. Practically in the whole history of biological theories there has been no simple fulminating discovery such as that known as the law of gravitation. Biologists are dealing with something that at present appears too complex to admit of simplification to this extent. Yet each biologist who begins applying his theories to sociology is undertaking a task a thousand times more difficult. The universal in zoology might be found, but the universal in sociology is not yet to hand. Some knowledge of philosophy, some real psychological insight must be allied to the zoologist's stock-in-trade. Herbert Spencer is considered by many of his more enthusiastic followers to have been such a genius, but it is questionable whether posterity will endorse this view.

After all, the ultimate touchstone of all experimentation must be its utility to man. Science for science's sake may be as noble an ideal as art for art's sake, yet science in its very essence must be capable of practical application. That many discoveries are made by men who are not looking for them has been true long before the Crusaders started to conquer Jerusalem and turned, as it were, aside to capture Constantinople. But not to everyone comes this good fortune. For the experimenter it is sufficient to have worked towards some notable end, provided, of course, that he has the wherewithal to live. Thus in biological experiments the greatest discoveries have been those which are associated with the names of Pasteur and Lord Lister. The practical application of these epoch-making discoveries is too well known, not only to all surgeons and physicians, but equally much so to all institutional workers, for one of their results has been a new method of hospital construction. It is far less easy to admit the practical utility of much modern research work in medicine, but one branch of it, bio-chemistry, certainly seems destined for great achievements. For all that, it is an unfortunate fact that hereditarians for the most part have entirely neglected the bio-chemical possibilities of heredity for miscroscopic work, which cannot be characterised by any other epithet than "kinder garten."

